# Clinicopathological Spectrum of Facial Granulomatous Dermatoses: Evidence from a 5-Year Study in Iran

**DOI:** 10.1155/2024/9946828

**Published:** 2024-06-01

**Authors:** Zeinab Aryanian, Mahshid Ansari, Huda Fatima, Mona Homayouni, Azadeh Khayyat, Alireza Ghanadan, Parvaneh Hatami

**Affiliations:** ^1^Autoimmune Bullous Diseases Research Center, Tehran University of Medical Sciences, Tehran, Iran; ^2^Department of Dermatology, Razi Hospital, Tehran University of Medical Sciences, Tehran, Iran; ^3^Department of Dermatology, Babol University of Medical Sciences, Babol, Iran; ^4^Blizard Institute, Queen Mary University of London, London, UK; ^5^Pathology Department of Medical College of Wisconsin, Milwaukee, WI, USA; ^6^Department of Dermatopathology, Razi Hospital, Tehran, Iran

## Abstract

**Background:**

Granulomatous dermatoses, particularly on facial skin, pose a diagnostic challenge, as similar histologic patterns can be produced by different causes.

**Aim:**

To evaluate the correlation between clinical suspicion and histopathological findings in various facial granulomatous dermatoses.

**Materials and Methods:**

This retrospective, cross-sectional study included all patients with the histopathological diagnosis of facial granulomatous dermatoses from the years 2016 to 2021 in an academic hospital. Demographic, clinical, and histopathologic features were reviewed and analyzed.

**Results:**

In this study, 150 histopathological records with the diagnosis of facial granulomatous dermatoses from the years 2016 to 2021 were reviewed. The most common clinical diagnosis was rosacea 34 (23.6%), followed by sarcoidosis 27 (18.8%), leishmaniasis 15 (10.4%), and granulomatous rosacea 10 (6.9%). The frequency of clinical diagnosis of rosacea (70.6), sarcoidosis (66.7), foreign body G (62.5), TB (75), pseudolymphoma (75), acne agminata (66.7), and granulomatous rosacea (70) in female patients was higher than that in males (*P* value = 0.03). The effect of age on the type of both clinical and histopathological diagnosis was statistically significant (*P* value = 0.0001 and 0.004, respectively).

**Conclusion:**

Our study contributed significantly to the understanding of the clinicopathological aspects of facial granulomatous dermatoses and advocated for a multidisciplinary approach to the diagnosis and management of these complex skin conditions.

## 1. Introduction

Granulomatous dermatoses, especially when presenting on the face, encapsulate a wide range of conditions distinguished by granuloma formation, a type of inflammation that is a hallmark response to various antigens. The intricate nature of these diseases, particularly those affecting the face, demands an in-depth understanding of their clinicopathological features to ensure accurate diagnosis and effective management. Distinguishing between granulomatous diseases requires careful evaluation of clinical symptoms and histopathological findings due to their overlapping features [1–3].

The complexities are amplified by the fact that such conditions can stem from a myriad of causes, including infections, autoimmune reactions, and foreign body responses, each presenting a unique challenge to healthcare professionals.

Advancements in diagnostic techniques, such as dermoscopy, have significantly improved our ability to diagnose and differentiate between various inflammatory dermatoses, including facial granulomatous disease (FGD). Sławińska and colleagues [4] have contributed to this area by systematically reviewing the role of dermoscopy in skin color, offering insights into its application for diagnosing inflammatory dermatoses. This highlights the evolution of diagnostic modalities in enhancing our understanding and management of these complex conditions.

Moreover, the intersection of FGDs with systemic diseases, as demonstrated by Yang et al.'s report [5] on systemic sarcoidosis manifesting with facial palsy and granulomatous reactions, further complicates the clinical picture. Such cases illustrate the systemic nature of granulomatous inflammations and the importance of considering a broader spectrum of differential diagnoses.

Compellingly, recent literature has also shed light on the coexistence of granulomatous conditions with other dermatological diseases, as seen in Afiouni et al.'s discussion [6] on severe granulomatous rosacea coexisting with cutaneous lupus erythematosus. This confluence of conditions underscores the intricate interplay between various dermatoses and highlights the critical need for a comprehensive diagnostic approach.

In this retrospective, cross-sectional study, we aim to delve into the clinicopathological spectrum of FGDs, drawing on a rich tapestry of cases diagnosed over five years in an academic hospital in Iran. By meticulously reviewing demographic, clinical, and histopathological features, this study seeks to illuminate the diverse etiological landscape of FGDs, advancing our understanding and management of these complex conditions. Through this endeavor, we contribute to the broader narrative on FGDs, offering insights that underscore the importance of a nuanced approach to diagnosis and treatment in the realm of dermatology.

## 2. Materials and Methods

To gather data, a meticulously structured two-part checklist was employed, covering both demographic information and histopathological details. This included recording the semiotics of the lesions (such as nodules, papules, plaques, and abscesses) and histologic characteristics such as the granuloma's placement relative to the dermis, alongside the patient's age, sex, and the specific location of the lesion, as extracted from pathological reports. Additional data on the patient's medical history (encompassing conditions such as thyroid disorders, diabetes mellitus, history of trauma, exposure to radiotherapy, and the presence of other skin lesions), the duration of the disease, medication history, changes in the epidermal layer, and the lesion's topography were gathered from medical records.

The protocol of this study was approved by the relevant ethics committee, and informed consent was obtained from all patients whose data was included in this study.

The study encompassed all cases of biopsied facial granulomatous dermatoses recorded in the pathology department of a hospital between 2016 and 2021, reflecting the condition's prevalence during these years.

For the analysis phase, the chi-square test was employed to explore the relationship between two categorical variables encompassing two or more categories, resorting to the Fisher exact test when deemed necessary. The association between a continuous variable and a categorical variable spanning more than two categories was evaluated using one-way ANOVA. Furthermore, Kappa statistics were utilized to assess the concordance between clinical and histopathological (HP) diagnoses. All statistical procedures were conducted using SPSS 26, with the threshold for statistical significance established at 5%.

## 3. Results

In this study, 150 histopathological records with the diagnosis of facial granulomatous dermatoses from the years 2016 to 2021 were reviewed; six patients with the confusing pathological diagnosis were excluded. To complete the data for 144 patients, pathology slides were again reviewed under a microscope by the dermatopathologist.

Based on our data, 61 (42.4%) of patients were male and 83 (57.6%) were female. The mean age of all patients in this study was 35.83 years, with a standard deviation of 21.01.

Some clinicopathologic features of tissue samples are summarized in Table 1.

The most common cutaneous findings were erythematous papulonodular 55 (38.2%), followed by erythematosus papulopustular 14 (9.7%), annular plaque with central atrophy 10 (6.9%), scar 3 (2.1%), abscess 3 (2.1%), indurate annular plaque with necrotic center 1 (0.7%), and 58 (40.3) cases with data not available. Histopathology of facial granulomatous lesions revealed 21.5% cases with tuberculoid necrotizing granuloma (31) followed by tuberculoid non-necrotizing granuloma 29 (20.1%), foreign body granuloma 23 (16%), sarcoid type granuloma 21 (14.6%), xanthogranuloma 10 (6.9%), nonspecific granuloma 8 (5.6%), actinic granuloma 4 (2.8%), granuloma annulare 4 (2.8%), necrobiotic type granuloma 3 (2.1%), sarcoid/foreign body like granuloma 1 (0.7%), sarcoid/tuberculoid type granuloma 1 (0.7%), lepromatous granuloma 1 (0.7%), pustular granuloma 1 (0.7%), tattoo granuloma 1 (0.7%), suppurative granuloma 1 (0.7%), and cases with data not available 5 (3.5%). Most commonly, the lesions were found throughout the dermis 59 (41%) and then confined to follicles throughout the dermis 20 (13.9%), upper to mid dermis 19 (13.2%), upper dermis 16 (11.1%), perifollicular/peri-infundibular 6 (4.2%), mid to deep dermis 2 (1.4%), and 22 (15.3%) with data not available. The frequency and percentage of biopsied facial granulomatous dermatoses on the basis of the presence or absence of inflammatory cells were as follows: plasma cell frequency (%) 57 (39.6), lymphocyte frequency (%) 127 (88.2), epithelioid histiocytic frequency (%) 116 (80.6), giant cell frequency (%) 82 (56.9), neutrophil frequency (%) 23 (16), eosinophil frequency (%) 14 (9.7), and fibroblast frequency (%) 1 (0.7). Epidermal changes, including atrophy, loss of rete ridge, acanthosis, papillomatosis, hyperkeratosis, and parakeratosis, were seen with the following percentages: atrophy/rete ridge effacement 28 (19.4%), spongiosis 8 (5.6%), acanthosis/hyperplasia 18 (12.5%), acanthosis and spongiosis 6 (4.2%), spongiosis and atrophy 3 (2.1%), Acanthosis and loss of rete ridges 1 (0.7%), detachment from dermis 1 (0.7%), erosion 1 (0.7%), keratin filled crypt invagination 1 (0.7%), papillomatous/lichenoid tissue pattern with melanin pigmentation 1 (0.7%), Thin epidermis, follicular plugging 1 (0.7%), trans-epidermal elimination 1 (0.7%), other 19 (13.2%), and data that was not available 55 (38.2%).

In the present study, the most common clinical diagnosis was rosacea 34 (23.6%), followed by sarcoidosis 27 (18.8%), Leishmaniasis 15 (10.4%), Granulomatous rosacea 10 (6.9%), acne agminata 9 (6.3%), foreign body Granuloma 8 (5.6%), TB 4 (2.8%), pseudolymphoma 4 (2.8%), Xanthogranuloma 4 (2.8%), Actinic granuloma 3 (2.1%), nevus3 (2.1%), and other dermatoses, including lichen planus, lymphoma, lupus vulgaris, morbihan edema, pilar cyst, acne rosacea, lupus miliaris, keratoacanthoma, cyst, scar, granuloma annular, DLE, SCC, and necrobiosis lipoidica.

The frequency of biopsied facial granulomatous dermatoses based on histopathological diagnosis was as follows: granulomatous rosacea 46 (31.9%), sarcoidosis 20 (13.9%), foreign body granuloma 16 (11.1%), acne agminata 8 (5.6%), xanthogranuloma 6 (4.2%), juvenile xanthogranuloma 4 (2.8%), tuberculoid G 11 (7.6%), leishmaniasis 7 (4.9%), pseudolymphoma 1 (0.7%), unremarkable 4 (2.8%), and others 21 (14.6%), including actinic granuloma, lupus miliaris, acne rosacea, benign lichenoid, granulomatous dermatitis, inclusion cyst, periorificial granulomatous dermatitis, BCC, neutrophilic (pustular) granuloma, granuloma annulare, non-necrotizing granulomatous dermatitis, ruptured inflamed inclusion cyst, rosacea, and necrobiosis lipoidica.

The frequency of clinical diagnosis of rosacea (70.6), sarcoidosis (66.7), foreign body G (62.5), TB (75), pseudolymphoma (75), acne agminata (66.7), and granulomatous rosacea (70) in female patients was higher than that in male patients, and this difference between gender and type of clinical diagnosis was statistically significant (*P* value = 0.03) (Table 2).

Regarding gender, the histopathologic diagnosis of xanthogranuloma (66.7), tuberculoid G (63.6), and leishmaniosis (71.4) in male patients was higher than that in female patients, but the difference was not statistically significant (*P* value = 0.21) (Table 2).

**Figure 1:**
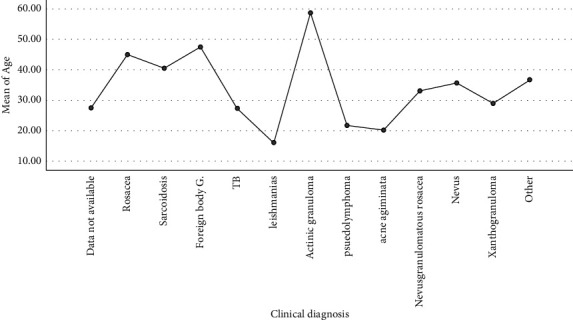
Mean plot of age according to the type of clinical diagnosis.

**Figure 2:**
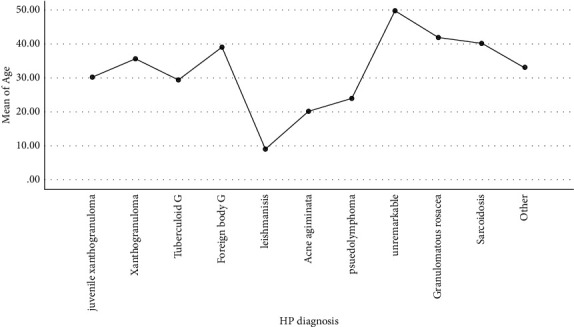
Mean plot of age according to the type of HP diagnosis.

According to Figure 1, the mean age of the patients with a clinical diagnosis of actinic granuloma and leishmaniasis were the highest and lowest, respectively (58.6 ± 13.6 and 16.1 ± 20.1, respectively), and the effect of age on the type of clinical diagnosis was statistically significant (*P* value = 0.0001). The significant effect of age was also noted on histopathologic diagnosis (Figure 2). The mean age of the patients with the HP diagnosis of granulomatous rosacea (41.8 ± 17.5) was the highest, and leishmaniosis (9.1 ± 16.2) was the lowest in this regard, and the effect of age on the type of HP diagnosis was statistically significant (*P* value = 0.004).

The clinicopathological concordance was brought in Table 3 for each dermatosis (Table 3).

## 4. Discussion

Facial granulomatous dermatoses pose diagnostic challenges due to their diverse clinical presentations and overlapping histopathological features. In this 5-year retrospective study conducted in Iran, we aimed to characterize the clinicopathological spectrum of facial granulomatous dermatoses and assess the agreement between clinical and histopathological diagnoses.

Our findings revealed a predominance of females (57.6%) among the studied patients, consistent with observations by Almutairi et al. [1] in their report on facial granulomatous rosacea. The mean age of patients in our study was 35.83 years, aligning with the demographic profile reported in similar investigations [7, 8].

Clinically, rosacea emerged as the most common diagnosis (23.6%), followed by sarcoidosis (18.8%) and leishmaniasis (10.4%). However, histopathological examination revealed granulomatous rosacea as the predominant diagnosis (31.9%), underscoring the importance of histopathological confirmation, as emphasized by Kok et al. [9] and Singh et al. [10].

Interestingly, while there was significant gender variation in clinical diagnoses, no such correlation was observed in histopathological diagnoses, echoing the findings of Afiouni et al. [6]. This highlights the complexity of diagnosing facial granulomatous dermatoses and suggests that histopathology remains the cornerstone for accurate diagnosis, particularly in cases with atypical clinical presentations.

Histopathological analysis unveiled a diverse array of granulomatous lesions, including tuberculoid necrotizing granuloma (21.5%) and sarcoid type granuloma (14.6%), consistent with the histopathological spectrum reported in studies by Dsouza et al. [3] and Rajbhandari et al. [11]. Furthermore, our study identified distinct patterns of epidermal changes, highlighting the importance of evaluating both dermal and epidermal features in diagnosing granulomatous dermatoses.

Moreover, our study provides valuable insights into the discrepancy between clinical and histopathological diagnoses. While rosacea was the most common clinical diagnosis, granulomatous rosacea was the predominant histopathological finding. This discordance underscores the necessity of integrating clinical, histopathological, and ancillary diagnostic modalities, such as dermoscopy [4], to achieve accurate diagnosis and guide appropriate management.

Notably, the correlation between age and diagnosis observed in our study aligns with previous reports emphasizing age-related variations in the presentation of granulomatous dermatoses [12]. Specifically, older patients were more likely to be diagnosed with granulomatous rosacea, whereas younger individuals exhibited a broader spectrum of diagnoses, including leishmaniasis and acne agminata.

In conclusion, our study provides valuable insights into the clinicopathological spectrum of facial granulomatous dermatoses in Iran, emphasizing the importance of a multidisciplinary approach for accurate diagnosis and optimal patient care. Further research is warranted to explore the underlying mechanisms driving the observed clinical and histopathological variations and refine diagnostic and therapeutic strategies for these complex dermatological conditions.

## Figures and Tables

**Table 1:** Clinicopathologic features of biopsied facial granulomatous dermatoses.

Type of granuloma	Frequency (%)	Epidermal changes	Frequency (%)	Semiology of the lesions	Frequency (%)	Location of dermis	Frequency (%)	Type of cells in the tissue sample	Frequency (%)
Foreign body granuloma	23 (16)	Atrophy/rete ridges effacement	28 (19.4)	Erythematous papulonodular	55 (38.2)	Upper dermis	16 (11.1)	Plasma cell	57 (39.6)
Tuberculoid non-necrotizing granuloma	29 (20.1)	Spongiosis	8 (5.6)	Erythematous papulopustular	15 (9.7)	Upper to mid dermis	16 (13.2)	Lymphocyte	127 (88.2)
Sarcoid type granuloma	21 (14.6)	Acanthosis/hyperplasia	18 (12.5)	Annular plaque with central atrophy	10 (6.9)	Mid to deep dermis	2 (1.4)	Epithelioid histiocyte	116 (80.6)
Tuberculoid necrotizing granuloma	31 (21.5)	Acanthosis and spongiosis	6 (4.2)	Scar	3 (2.1)	Perifollicular/peri-infundibular	6 (4.2)	Giant cell	82 (56.9)
Xanthogranuloma	10 (6.9)	Spongiosis and atrophy	3 (2.1)	Abscess	3 (2.1)	Throughout the dermis	59 (41)	Neutrophil	23 (16)
Nonspecific granuloma	8 (5.6)	Acanthosis and loss of rete ridges	1 (0.7)	Indurate annular plaque with necrotic center	1 (0.7)	Confined to follicle throughout the dermis	20 (13.9)	Eosinophil	14 (9.7)
Actinic granuloma	4 (2.8)	Detachment from dermis	1 (0.7)					Fibroblast	1 (0.7)
Granuloma annulare	4 (2.8)	Erosion	1 (0.7)						
Necrobiotic type granuloma	3 (2.1)	Keratin filled crypt invasgination	1 (0.7)						
Sarcoid/foreign body like granuloma	1 (0.7)	Papillomatous/lichenoid tissue pattern with melanin pigmentation	1 (0.7)						
Sarcoid/foreign body type granuloma	1 (0.7)	Thin epidermis/follicular plugging	1 (0.7)						
Lepromatous granuloma	1 (0.7)	Trans-epidermal elimination	1 (0.7)						
Pustular granuloma	1 (0.7)	Other	19 (13.2)						
Tattoo granuloma	1 (0.7)								
Suppurative granuloma	1 (0.7)								
Data not available	5 (3.5)		55 (38.2)		58 (40.3)		22 (15.3)		
Total	144 (100)		144 (100)		144 (100)		144 (100)		

**Table 2:** Relationship between sex and clinical/HP diagnosis by the chi-square test.

Diagnosis	Male *N* (%) (clinical diagnosis)	Female *N* (%) (clinical diagnosis)	Male *N* (%) (HP diagnosis)	Female *N* (%) (HP diagnosis)
Data not available	4 (100)	0	—	—
Rosacea	10 (29.4)	24 (70.6)	—	—
Sarcoidosis	9 (33.3)	18 (66.7)	5 (25)	15 (75)
Foreign body G.	3 (37.5)	5 (62.5)	8 (50)	8 (50)
TB	1 (25)	3 (75)	—	—
Leishmaniosis	10 (66.7)	5 (33.3)	5 (71.4)	2 (28.6)
Actinic granuloma	3 (100)	0	—	—
Pseudolymphoma	1 (25)	3 (75)	—	—
Acne agminata	3 (33.3)	6 (66.7)	3 (37.5)	5 (62.5)
Unremarkable	—	—	1 (25)	3 (75)
Granulomatous rosacea	3 (30)	7 (70)	15 (32.6)	31 (67.4)
Nevus	2 (66.7)	1 (33.3)	—	—
Xanthogranuloma	1 (25)	3 (75)	4 (66.7)	2 (33.3)
Juvenile xanthogranuloma	—	—	2 (50)	2 (50)
Tuberculoid G	—	—	7 (63.6)	4 (36.4)
Others	11 (57.9)	8 (42.1)	11 (52.4)	10 (47.6)
Total	61 (42.4)	83 (57.6)	61 (42.4)	83 (57.6)

Fisher exact test = 20.3, *P* value = 0.03 for clinical diagnosis. Fisher exact test = 12.7, *P* value = 0.21 for HP diagnosis.

**Table 3:** Agreement between clinical and HP diagnosis.

	Both positive/total HP positive	Both negative/total HP negative	Kappa statistics	Agreement
Sarcoidosis	18/20	115/124	0.72	Substantial
Foreign body granuloma	8/16	127/128	0.61	Substantial
Pseudolymphoma	1/1	140/143	0.39	Fair
Acne agminata	8/8	134/136	0.88	Favorable
Granulomatous rosacea	8/46	97/98	0.21	Fair
Xanthogranuloma	4/10	134/134	0.55	Moderate

## Data Availability

The data used to support the findings of this study are available from the corresponding author upon reasonable request.
